# Thrombotic microangiopathy with irreversible renal function in a CKD patient following COVID-19: case report

**DOI:** 10.3389/fmed.2025.1484385

**Published:** 2025-09-18

**Authors:** Yunlong Qin, Xinjun Yang, Wei Zhao, Yuzhe Li, Yaling Bai, Lihui Wang

**Affiliations:** ^1^Department of Nephrology, Bethune International Peace Hospital, Shijiazhuang, China; ^2^Department of Nephrology, The Fourth Hospital of Hebei Medical University, Shijiazhuang, China

**Keywords:** COVID-19, thrombotic microangiopathy, acute kidney injury, chronic kidney disease, CRRT

## Abstract

We report a case of chronic kidney disease (CKD), in a patient with normal renal function, diagnosed as acute renal failure stage 3 after coronavirus disease 2019 (COVID-19)-associated thrombotic microangiopathy (TMA) characterized by the presence of thrombocytopenia, microangiopathic hemolytic anemia, and acute renal failure. The results of the renal biopsy were thickening of the small artery wall with mucous edema, endothelial swelling and hyperplasia, and segmental endothelial onion-like hyperplasia. However, the pathogenesis factors of the patient who suffered from COVID-19-associated TMA were not clear, although blood complements, activity of ADAMTS13, and Factor H were detected as normal. After the administration of pulse glucocorticoid, plasma exchange, and hemodialysis, the patient developed chronic renal failure and depended on peritoneal dialysis 12 months later. Although the clinical manifestations of TMA are similar, the etiology and pathogenesis are very complex. It is very important to determine the pathogenesis of TMA to proceed with precise treatment to improve the prognosis of the patient.

## 1 Introduction

Thrombotic microangiopathy (TMA) is a group of disorders characterized by the presence of thrombocytopenia, microangiopathic hemolytic anemia (MAHA), and end-organ capillary thromboses that result from microthrombi in capillaries and arterioles. The incidence of acute kidney injury (AKI) induced by COVID-19 infection has significantly increased due to various reasons, with multiple cases manifesting as TMA, which is more prevalent in severe cases and autopsies (1). Severe acute respiratory syndrome coronavirus 2 (SARS-CoV-2) can cause TMA via cellular toxicity, complement activation, immune dysregulation, and coagulopathy immune dysregulation (2). We hereby describe a case of TMA caused by SARS CoV-2 infection in a patient who exhibited proteinuria with normal renal function 8 months prior. Despite the administration of pulsatile corticosteroid therapy, plasma exchange, and continuous veno-venous hemodiafiltration (CVVHDF), renal function failed to recover in this patient. It is very important to determine the pathogenesis and clinical type of TMA to provide precise treatment for patients with COVID-19-associated TMA. The study acquired proper institutional approval (2023-KY-179) and necessary consent from the patient.

## 2 Case presentation

### 2.1 Investigation, diagnosis and subsequent treatment

**FIGURE 1:**
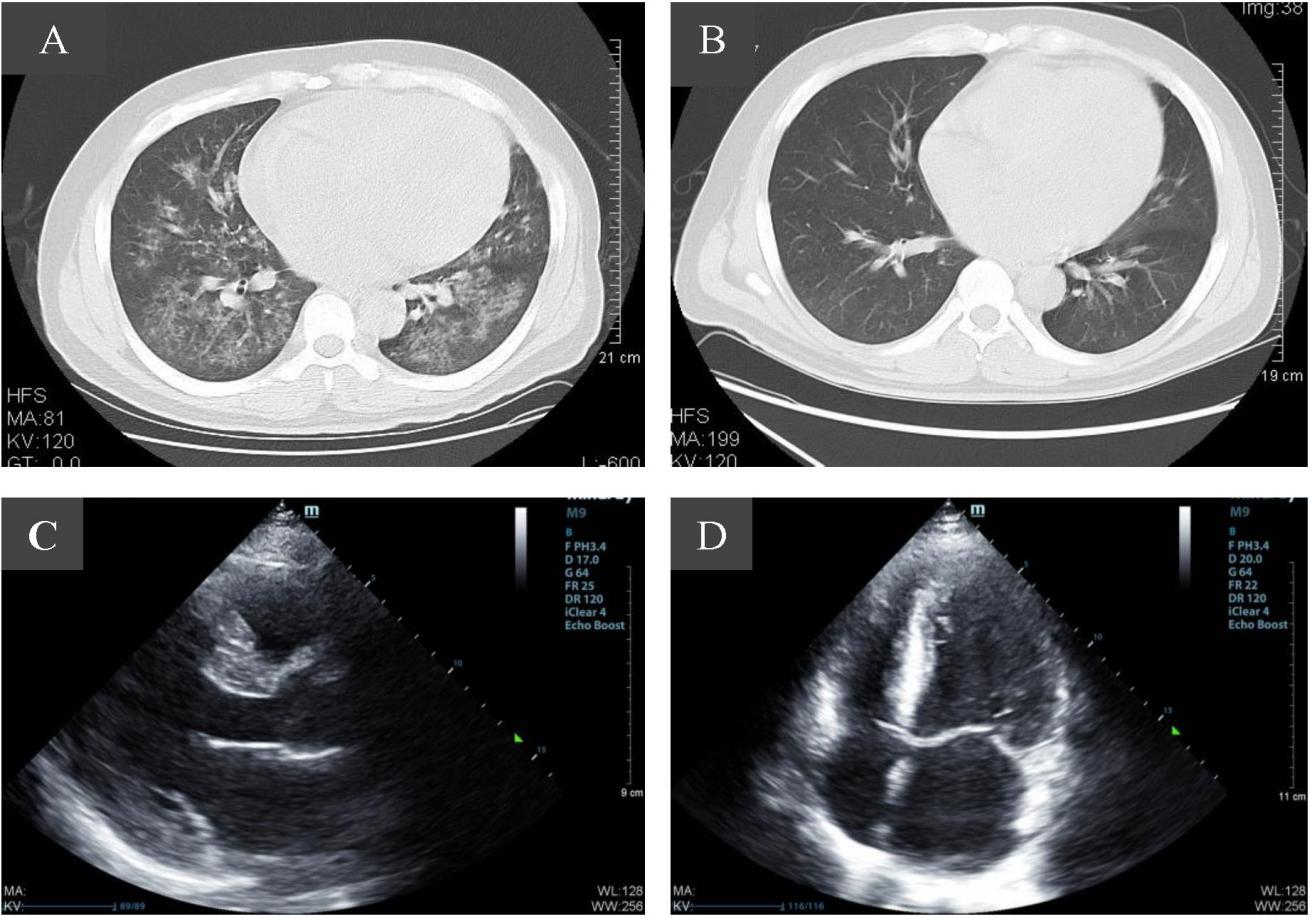
Imaging changes of patients before and after admission. **(A)** Pulmonary CT scan at admission showed evidence consistent with viral pneumonia combined with pulmonary edema. **(B)** Ten days after admission, pulmonary infection was significantly reduced. **(C,D)** Color Doppler echocardiography showed an enlarged heart size with a large left atrium and left ventricular hypertrophy.

A 34-year-old Asian male was admitted to our hospital emergency due to intermittent hematuria for 2 weeks, nausea for 5 days, and expiratory dyspnea for 3 days. The patient was found with proteinuria with hypertension 150/90 mmHg and normal renal function 8 months ago, but did not do further examination. He suffered from COVID-19 2 weeks before and was not taking any medication. After admission, a physical examination indicated he was in a sitting position with a rapid heart rate, wet rales in both lungs and symptoms of acute left heart failure. His blood pressure was 160-170/90-100 mmHg, and the chest computed tomography (CT) scan showed patchy and flaky hyperdense shadows in both lungs, partially fused into a large area, with enlarged cardiac shadows, consistent with viral pneumonia, combined with pulmonary edema (Figure 1A). Color Doppler echocardiography showed an enlarged heart size with a large left atrium and left ventricular hypertrophy (Figures 1C, D). Renal ultrasound: both kidneys were normal in size, with increased cortical echogenicity of the kidneys. Clinical investigations revealed anemia, thrombocytopenia and AKI. The serum creatinine (Scr) level was 1417 μmol/L associated with 0.5 g per day proteinuria, lactate dehydrogenase 2423 U/L, hemoglobin 53 gL and platelets 33 G/L (Table 1), with negative direct anti-human globulin test. The nucleic acid test and IgM antibody of SARS-CoV-2 were positive. The patient tested negative for antinuclear antibody (ANA), anti-double-stranded DNA (dsDNA), anti-SSA, anti-SSB antibodies, rheumatoid factor, MPO-ANCA, and PR3-ANCA, ruling out diseases such as systemic lupus erythematosus, Sjögren’s syndrome, and ANCA-associated vasculitis. There was no evidence of post-renal obstructive disease, paraproteinemia, or malignancy, and the patient had no history of diabetes or heart disease. Gastrointestinal symptoms such as nausea, vomiting, and diarrhea after virus infection. The patient was diagnosed with acute kidney injury stage 3, hemolytic uremic syndrome, pulmonary edema, and acute left heart failure.

**TABLE 1:** Changes of the major clinical parameters of the index patient.

Characteristics	Reference range	Course of disease									
		Upon admission	1 W	2 W	4 W	8 W	12 W	16 w	24 W	28 w	56 w
Urinary output, ml/24 h	1500–2500	350	400	750	1200	2200	1500	1900	2950	2350	2430
Platelet, G/L	125–350	33	86	82	118	176	186	151	147	153	234
Hemoglobin, G/L	130–175	53	80	72	81	79	81	140	110	97	115
Creatinine, umol/L	57–97	1417	846	916	834	801	832	759	501	519	320
LDH, U/L	120–250	1803	460	369	326	381	252	200	210	209	286
Myoglobin, ng/ml	0–90	891	643	359	149	112	59	65	67	167	89
D-Dimer, mg/L	0–0.243	0.424	0.449	N/A	N/A	2.190	4.09	1.232	0.822	0.432	0.241
NT-Pro BNP, pg/ml	0–250	45000	4030	12873	N/A	3415	2554	N/A	N/A	N/A	818
UTP, mg/24 h	20–141	500	345	N/A	432	453	344	465	432	345	59

W, week; LDH, lactate dehydrogenase; NT-pro BNP, N-Terminal pro-Brain Natriuretic Peptid; UTP, total urinary protein; N/A, not available.

On the second day, the patient exhibited a 24-h urine volume of approximately 350 milliliters, and the symptoms of acute left heart failure persisted despite intervention. Consequently, CVVHDF was administered. Pulse therapy using methylprednisolone was started expeditiously, 500 mg daily for 3 days, followed by 40 mg once daily. Sodium nitroprusside was administered intravenously to control blood pressure, deslanoside injection was used to correct heart failure, cefoperazone sodium and sulbactam sodium were employed for anti-infectious treatment, packed red blood cells were transfused to correct anemia, and esomeprazole sodium for injection was used to inhibit gastric acid secretion and protect the gastric mucosa. Notably, during the initial disease phase, therapeutic administration of eculizumab was precluded by both the patient’s financial constraints and limited local drug availability. Consequently, an intensified plasma exchange protocol was implemented, comprising six alternate-day sessions with 2,000 mL plasma volume exchange per session.

On the third day, complement C3, C4, activity ADAMTS13 and complement factor H were normal. On the seventh day, the 24-h urine output was still 400 ml, while the syndrome of heart failure improved. On the 10th day, a pulmonary CT scan showed scattered patchy slightly hyperdense shadows in both lungs with blurred margins (Figure 1B). The blood pressure gradually decreased to 140-150/80-90 mmHg. On the 30th day, platelets gradually returned to normal, the urine output gradually increased to 1300 ml/24 h, and the Scr was 916 umol/L.

**FIGURE 2:**
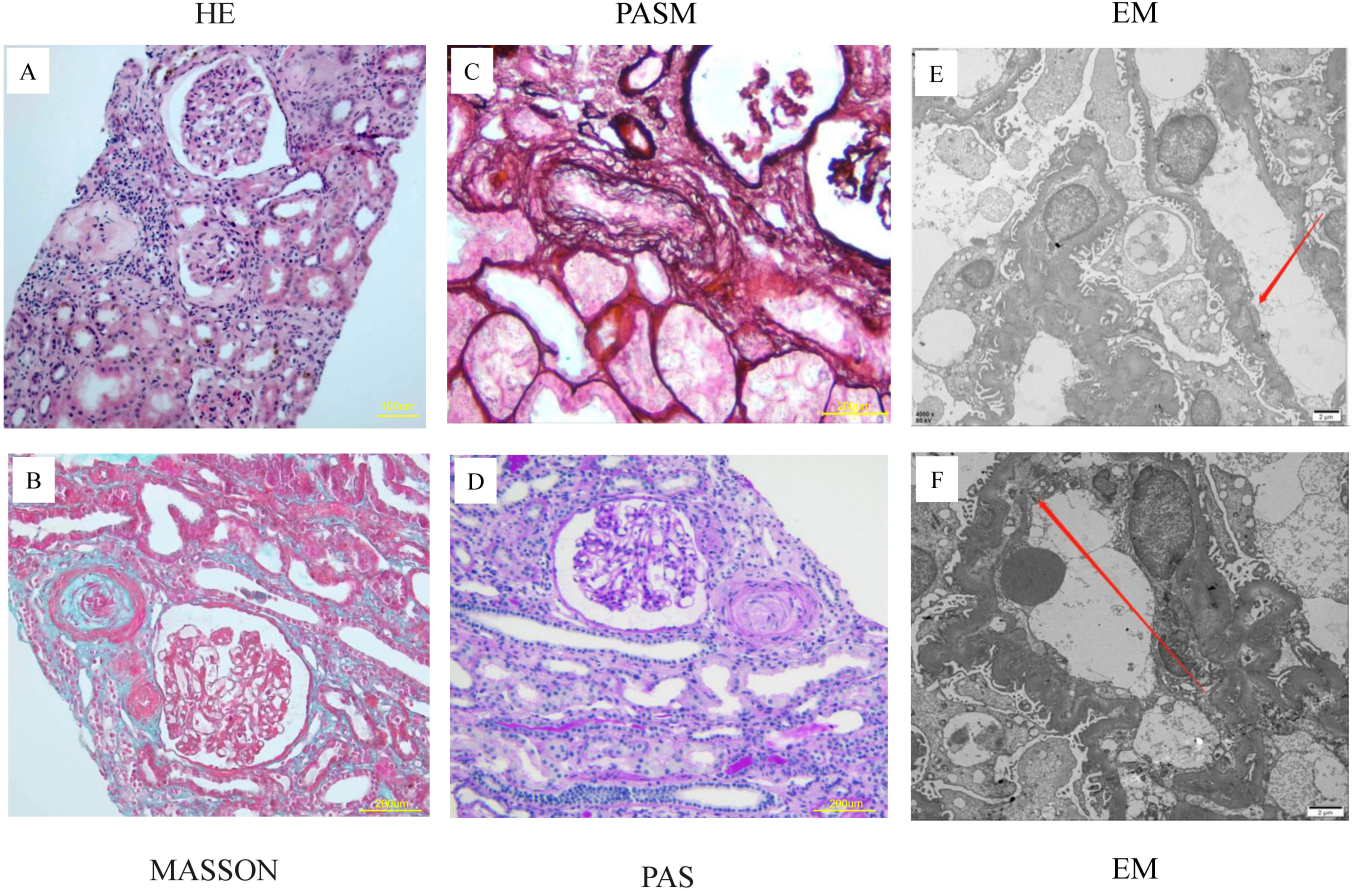
Pathological features of renal biopsy in the index patient. **(A–D)** Renal pathology seen in the light microscope: 20 glomeruli, 5 of which had ischemic sclerosis, no platelets or fibrin thrombosis were seen in the glomeruli. The glomerular capillary loops were poorly opened and partially crumpled with ischemic glomeruli. The wall of small arterioles was thickened with mucous edema and endothelial swelling and hyperplasia, segmental intimal onion-like hyperplasia, the segmental tubular wall was vitrified, and the lumen was narrowed. **(E,F)** Electron microscopy: ischemic crumpling of the glomerulus, widening of the segmental subendothelial space, and partial fusion of the peduncle.

The patient received renal biopsy, and pathology revealed the wall of small arterioles was thickened with mucous edema and endothelial swelling and hyperplasia, segmental intimal onion-like hyperplasia, the segmental tubular wall was vitrified, and the lumen was narrowed. The epithelial cells of the renal tubules were granularly degenerated and focally atrophied. No platelets or fibrin thrombosis were seen in the glomeruli. The renal pathological changes of the patient were dominated by the manifestations of small arterial TMA, ischemic shrinkage of the glomeruli and widening of the subendothelial gap (Figure 2). The immunofluorescence results showed negative staining for IgG, IgA, and IgM, with weak positive granular deposition of C3 in the glomerular basement membrane.

**FIGURE 3:**
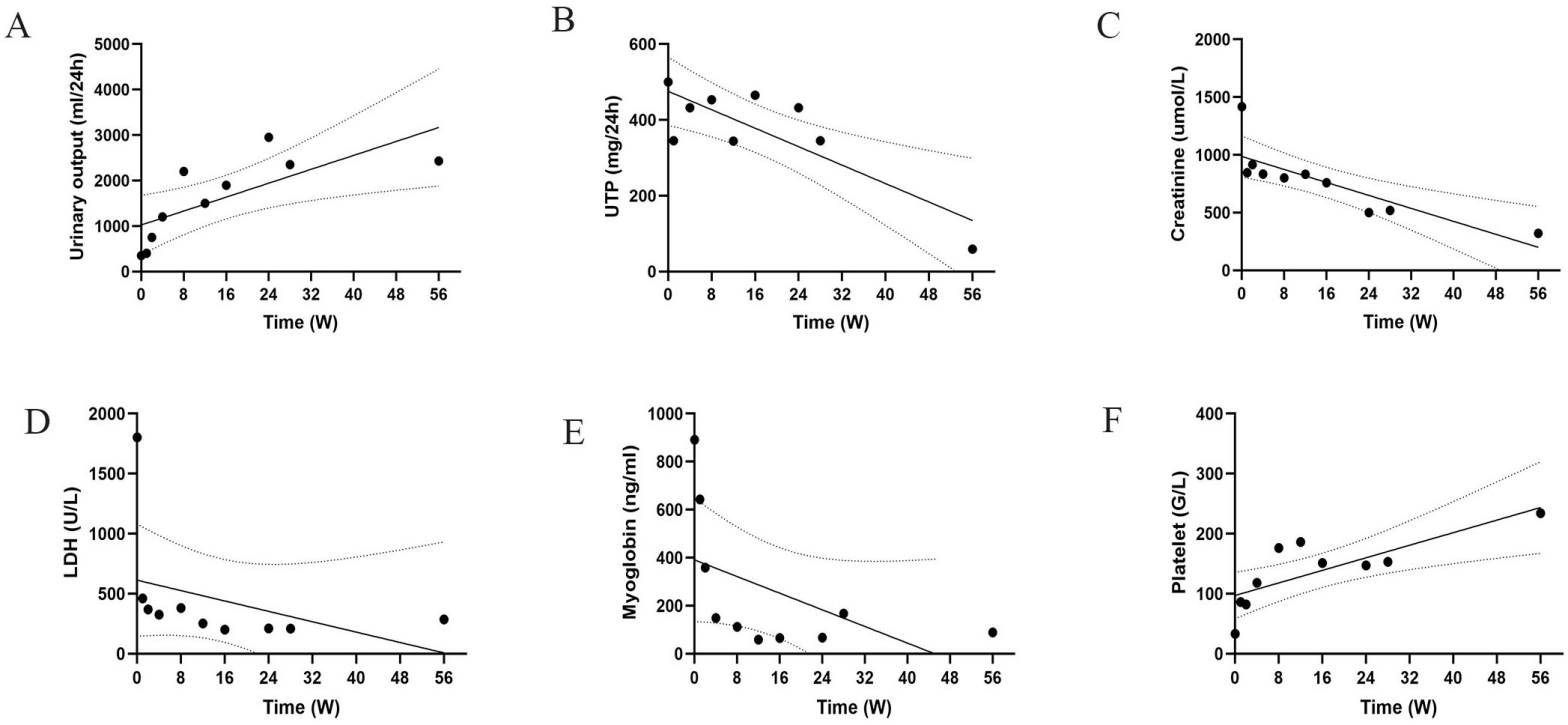
Trend of indicators during the patient’s disease course. **(A–F)** Time variation trends of different key indicators on a weekly basis. Each chart shows data points, trend lines and confidence intervals. UTP, total urinary protein. LDH, lactate dehydrogenase.

On the 90th day, the patient’s urine output returned to normal and dialysis was changed to peritoneal dialysis. Unfortunately, he became peritoneal dialysis dependent. At 6 months, the patient resumed normal activities of daily living with an Scr of 519 μmol/L. At week 56, the Scr was 320 μmol/L with 59 mg per day of proteinuria (Figure 3). The frequency of peritoneal dialysis was gradually reduced to 3 bags of dialysis fluid per day, 5 days per week. The anticoagulant drug urokinase (200 000 U) was given intravenously for 10 days per month (3). In addition, endothelial cell protective agents such as beraprost sodium and sulodexide were administered orally daily.

## 3 Discussion

Acute kidney injury is common among hospitalized patients with COVID-19, with the occurrence of AKI ranging from 0.5 to 80%. The incidence of AKI in patients admitted to the ICU for respiratory failure is 96% (4). The incidence of renal injury in the form of TMA in native kidneys is high (5). Multiple factors are intricately associated with TMA, encompassing medications, malignancies, genetic aberrations, and infectious diseases. Classically, hemolytic uremic syndrome (HUS) and thrombotic thrombocytopenic purpura (TTP) are the clinical syndromes of TMA (6). The renal biopsy definitively confirmed the manifestation of TMA, thereby leading to an unequivocal diagnosis of the condition. Due to the lack of complement gene testing, the exact etiological factors underlying the patient’s development of COVID-19-associated TMA remain unclear. After the administration of pulse hormones, plasma exchange, and hemodialysis, the patient remained dependent on peritoneal dialysis 12 months later. Thus, the present case is characterized by a patient with CKD and normal renal function who become dependent on renal replacement treatment after SARS-CoV-2 infection. To further identify the pathogenesis and clinical type of TMA and provide precise treatment is very important.

The most common comorbidities associated with COVID-19-related TMA were diabetes mellitus (DM), hypertension (HT), heart disease and CKD (7). The patient tested positive for SARS-CoV-2 nucleic acid accompanying nausea and vomiting gastrointestinal symptoms 2 weeks before he went to hospital. After hospitalization, the chest CT scan for the patient showed viral pneumonia caused by SARS-CoV-2. TMA occurs 2 weeks after the patient’s infection. Infection with COVID-19 resulted in almost no or only mild respiratory symptoms in the majority of patients, while digestive symptoms occurred in almost one-third of patients. However, it was observed that patients exhibiting digestive symptoms tend to present for treatment later compared to those with respiratory symptoms, suggesting that TMA with gastrointestinal manifestations may arise after the clearance of viremia (8). Despite the detection of proteinuria 8 months prior, renal function remained within normal limits, leading to the consideration of COVID-19 as a potential triggering factor for the development of TMA.

The pathological changes in TMA caused by COVID-19 mainly involve glomeruli and arterioles, with swelling of glomerular capillary endothelial cells and widening of the subendothelial cell gap (9). The pathological findings of TMA in this patient were arteriolar wall thickening with myxedema, endothelial cell swelling and proliferation, and segmental onion-like endothelial cell proliferation. These findings suggest the prior proteinuria likely stemmed from renal vascular sclerosis and hyaline degeneration, with hypertension serving as the probable causative factor. However, renal biopsy remains exceptionally limited and precious, given that most patients with AKI post-COVID-19 infection do not undergo this diagnostic procedure (10). In patients undergoing diagnostic kidney biopsy, the incidence rates of TMA in the glomerulus and vasculature were observed to be 9 and 8%, respectively. In contrast, postmortem kidney biopsy studies revealed a slightly higher prevalence of TMA in glomerular and vascular tissues, with rates of 10 and 12%, respectively (11). Therefore, even if the patient has been clinically diagnosed with TMA, it is necessary to clarify the renal pathology by renal biopsy. Renal biopsy can not only clarify the severity of the patient’s condition but also guide clinical treatment.

Although COVID-19-associated TMA disorders share many similarities in clinical presentation, the underlying pathophysiology is completely different and requires a specific approach (12). Direct viral toxicity to the vascular endothelium, immune dysregulation, a hyperinflammatory state and activation of the complement system may related to the pathogenesis of COVID-19-related TMA. These factors lead to endothelial damage followed by the release of substances such as VEGF and PDGF, which cause microvascular thrombosis (13). An ADAMTS13 activity level of less than 10% supports a diagnosis of TTP. Atypical HUS (aHUS) is defined as TMA caused by activation of the complement pathway (14). The aHUS can be either primary/hereditary with uncontrolled activation of the alternative complement pathway. Uncontrolled complement pathway activation may play a role in the pathogenesis of aHUS. Genetic mutations in complement pathway regulators result in the formation of membrane attack complexes (C5b-9), which lead to renal endothelial injury, coagulation cascade activation and microthrombosis of small renal arteries (15). In addition, antiphospholipid antibody syndrome and decreased adam13 activity all play a role in COVID-19-related TMA (16). The index patient clinically presented with hematuria, microvascular hemolytic anemia, acute renal failure, thrombocytopenia, and elevated LDH, which are typical symptoms of HUS. The normal ADAM13 activity excludes TTP. Acute vasculitis flares accompanied by C3 deficiency are often associated with multisystem involvement of other organs such as the lungs, nose, ears, and skin. Renal manifestations can include hematuria, proteinuria, concurrent acute kidney injury, and anemia, although platelet counts are typically normal. Renal pathological changes include focal and segmental necrosis of glomerular capillary loops and crescent formation, with oligoimmune complexes often observed on immunofluorescence. In contrast, patients with COVID-19 infection and reduced C3 levels tend to present with more severe clinical manifestations and a higher incidence of multiple organ dysfunction syndrome (17). These patients exhibit higher levels of inflammatory markers such as ferritin and IL-6, as well as elevated D-dimer levels, which are associated with increased disease severity and mortality. In this case, no evidence of complement activation was found during the hospital stay, and normal complement C3 and C4, and normal complement H factor were detected for the patient. Unfortunately, due to the unavailability of complement mutation analysis in our clinical laboratory, we refrained from conducting further diagnostic evaluations, including mutation testing. The treatment of COVID-19-associated TMA is decided based on the etiology and pathogenesis of the patient. Glucocorticoids, plasma exchange, anticomplement therapy, and anticoagulation therapy are the main treatments (18). Glucocorticoids can be effective in COVID-19-associated TMA by inhibiting ADAMTS13 and anti-Factor H antibodies, reducing endothelial cell inflammation, increasing endothelial carbon monoxide synthase activity, increasing NO synthesis, attenuating platelet aggregation, and blocking complement bypass pathway activation (19). Plasma therapy, including plasma exchange (PE) and plasma infusion (PI), can be promptly initiated, especially in the case of aHUS caused by mutations in the gene for complement regulatory protein of the complement paracrine pathway or caused by a positive antibody to the anti-H-factor, or in the case of TTP with decreased activity of ADAM13 (20). Recombinant humanized anti-C5 monoclonal antibodies can effectively improve the abnormal activation of the complement system, and its efficacy and safety have been reported (21). Gupta et al. found that female patients with first-episode pregnancy-related aHUS who received eculizumab treatment had significantly better outcomes compared to those who did not receive eculizumab (22). Additionally, eculizumab is a successful treatment option for pediatric patients with Ahus (23). In a patient diagnosed with TMA after ARS-CoV-2 infection, complement C3 and C4 were significantly lower than normal, but Factors H and B were in the normal range. Conventional therapeutic strategies, including high-dose steroids and seven sessions of therapeutic plasma exchange, were all unsuccessful. However, the application of eculizumab as a rescue therapy led to rapid clinical improvement and amelioration of laboratory indicators (24). This patient presented with acute disease onset. Due to patient-related factors, eculizumab therapy was not administered. As alternative therapy, we implemented pulse hormone therapy 500 mg for 3 days and six sessions of plasma exchange and continuous vein-vein hemodialysis filtration; 1 year later, his renal function had recovered partly, and he remained dependent on peritoneal dialysis. Once the patient was diagnosed with COVID-19-related TMA, even if there was no evidence to support complement activation, pulse glucocorticoid therapy, plasma exchange and CRRT were necessary to save the life of the patient. Once the evidence of activation of the complement bypass pathway was established, eculizumab should be applied immediately. Such evidence includes complement component and regulatory factor (CFH, C3, CFI, MCP, C4, and CFB) activity markers (CH50 and AH50), anti-CFH autoantibodies, and genetic screening for genes associated with aHUS (CFH, CFI, MCP, C3, CFB, THBD, DGKE, and CFHR) (25).

The prognosis of TMA patients depends on many factors, including black race, concomitant disease, the presence of CKD, and the nature of the complement mutation (26). Regarding outcomes of the patients with COVID-19-related TMA, 8.2% of patients remained dependent on HD after discharge (27). More African Americans had a higher rate of accepting kidney replacement therapy than other races and had worse outcomes with COVID-19 than others. Our present case exhibits multisystem damage, including viral pneumonia, heart failure, renal failure complicating chronic kidney disease, and hypertension. Despite hospital admission and treatment with steroid pulse therapy, plasma exchange, hemodialysis, and other interventions, the preexisting chronic kidney injury has become more irreversible.

## 4 Conclusion

COVID-19-related TMA has a poor prognosis. It is necessary to explore the pathogenesis of COVID-19-related TMA promptly and choose the corresponding treatment. Once TMA is diagnosed, early identification of the TMA subtype and appropriate prompt and specific treatment could improve survival and cure statistics for TMA of all causes. Even if the pathogenesis factors are not identified, treatments such as hormones and plasma exchange are necessary. Renal biopsy helps to determine the diagnosis and the prognosis of patients with TMA and is performed as soon as possible.

## Data Availability

The raw data supporting the conclusions of this article will be made available by the authors, without undue reservation.
